# A Microstructure Insight of MTA Repair HP of Rapid Setting Capacity and Bioactive Response

**DOI:** 10.3390/ma13071641

**Published:** 2020-04-02

**Authors:** María Carmen Jiménez-Sánchez, Juan José Segura-Egea, Aránzazu Díaz-Cuenca

**Affiliations:** 1 Department of Stomatology, Faculty of Dentistry, University of Sevilla, 41009 Sevilla, Spain; jimenezsanchez6@gmail.com (M.C.J.-S.); segurajj@us.es (J.J.S.-E.); 2 Materials Science Institute of Seville (ICMS), Joint CSIC-University of Sevilla Center, 41092 Sevilla, Spain; 3 Networking Research Center on Bioengineering, Biomaterials and Nanomedicine (CIBER-BBN), 28029 Madrid, Spain

**Keywords:** bioactive endodontic cement, calcium silicate-based materials, MTA Repair HP, microstructure, SBF, bioceramic characterization

## Abstract

Mineral trioxide aggregate (MTA) is considered a bioactive endodontic material, which promotes natural mineralization at the material-tooth tissue interface. MTA Repair HP stands out because of the short setting time and the quick and effective bioactive response in vitro. The bioactivity, depens on material composition and microstructure. This work is devoted to analyze MTA Repair HP microstructural features, of both the powder precursor and set material, to get insights into the material physicochemical parameters—functionality performance relationships. Transmission electron microscopy (TEM), and field emission gun scanning electron microscopy (FEG-SEM) coupled with energy-dispersive X-ray (EDX) analyses were performed. X-ray diffraction (XRD) measurements were carried out at different times to investigate setting process. Bioactivity evaluation in vitro was carried out by soaking the processed cement disk in simulated body fluid (SBF). The presented results point out those MTA Repair HP precursor material characteristics of tricalcium silicate particles of nanometric size and high aspect ratio, which provide an elevated surface area and maximized components dispersion of calcium silicate and very reactive calcium aluminate. The MTA Repair HP precursor powder nanostructure and formulation, allows a hydration process comprising silicate hydrate structures, which are very effective to achieve both fast setting and efficient bioactive response.

## 1. Introduction

One main property of the bioactive endodontic cements (BECs) is the capability to promote calcium phosphate (CaP) mineralization processes, which allows stable interfacial layer formation between the cement and teeth living tissues [1,2]. The bioactivity, as a promotion of the interfacial mineralization process, is a consequence of the biomaterial reactivity in contact with phosphate-containing physiological fluids [3,4]. An artificial material can bind to living tissue by the formation of a biomimetic carbonated hydroxyapatite layer, being recognized as natural for the biological and cellular environment [5,6].

Mineral trioxide aggregate (MTA), mainly comprised of calcium silicates, is a BEC commonly used in multiple dental therapeutic applications [7]. As pulp-capping material, hydrated MTA interact with dentine to induce the formation of the interfacial layer and the intratubular mineralization process [8], which not only influence the push-out bond strength but also stimulate repair and dentinogenesis or cementogenesis [9,10]. The setting reaction of MTA produces calcium silicate hydrate and calcium hydroxide, exhibiting high alkalinity and Ca ions release, which is considered to be a key mechanism behind the biocompatibility and bioactivity [11,12].

Current MTAs have limitations of slow setting rate and granular consistency, due to their high crystallinity and large particle sizes with irregular shapes [13,14,15]. Bioactivity, as a surface phenomenon largely deepens on material composition and microstructure [16,17]. In this sense, the control of the biomaterial physicochemical parameters may allow to regulate the bioactive response and therefore the capacity to stimulate and promote an adequate biological and cellular response for regeneration [18].

The new MTA Repair HP material stands out as a singular BEC material because of both, the short setting time and the quick and effective bioactive response in vitro [19]. MTA Repair HP has shown cytocompatibility and the ability to promote an adequate biological response in human dental pulp stem cells (hDPSCs) in terms of cell proliferation, morphology, migration, and attachment [20].

Previous work, comparing MTA Repair HP physicochemical parameters with those of ProRoot MTA White and NeoMTA Plus, carried out by our group has reported HP distinctive physicochemical parameters [21]. Particularly, un-hydrated MTA Repair HP in the powder form shows characteristic elongated nanoparticles within 50–100 nm range size thickness [22], which we associate to the high BET surface area of 4.8 m^2^ g^−1^ for this material. Apart from use of different radiopacifying components, CaWO_4_, Bi_2_O_3_, and Ta_2_O_5_ for MTA Repair HP, ProRoot and NeoMTA, respectively, the relatively high aluminum content and the absence of compositional sulphate phases are other identified MTA Repair HP material specific characteristics. All these concomitant features are likely to influence MTA Repair HP fast setting time and high bioactive response.

The focus of this paper is to analyze further the microstructure of both powder precursor and set material using electron microscopy and X-ray diffraction techniques. In this work, energy dispersive spectroscopy chemical analysis and high resolution transmission electron microscopy are used to investigate crystal phase components morphology and their relative distribution for un-hydrated precursor. Hydration products formation relative to the setting time process are studied using X-ray diffraction and field emission scanning electron microscopy.

## 2. Materials and Methods

MTA Repair HP (Angelus, Londrina, Brazil–lot n. 38585) of chemical composition specifications of tricalcium silicate (Ca_3_SiO_5_), dicalcium silicate (Ca_2_SiO_4_), calcium tungstate (CaWO_4_), tricalcium aluminate (Ca_3_Al_2_O_6_), and calcium oxide (CaO) were used in this study. For the hydrated material preparation, the powder was mixed according to manufacturer’s instructions with Milli-Q water only, to avoid the influence of specific product manufacturer additives, and to analyze then specific ceramic powder compositional properties. The manual mixing was performed adding the liquid to the powder on a glass slab, and the cement was blended using a metal spatula. A paste with homogeneous consistency was obtained. The paste was compacted in a silicone mold of 10 mm in diameter and 4 mm high and allowed to set for various time intervals at 37 °C and 95% relative humidity.

### Materials Characterization

Transmission electron microscopy (TEM) micrographs and energy-dispersive X-ray (EDX) analyses were obtained using a JEOL-2100 plus with a LaB6 filament as the electron source operated at 200 kV coupled to an X-ray dispersive energy analysis system (EDX X-Max 80 T, Oxford Instruments, Oxford, UK), and a CCD (Gatan) camera for image recording. Observations and analysis were performed for both un-hydrated and set material in the powder form, after previous material particles dispersion in ethanol. The set material was previously gently grinded. A drop of the prepared dispersions was deposited on a copper TEM grid covered by a carbon film. Field emission gun scanning electron microscopy (FEG-SEM) observations were performed using a HITACHI S-4800 (Tokyo, Japan). Images were recorded at an accelerating voltage of 2 kV. Energy dispersive X-ray (EDX) analysis was carried out at 10 kV with an EDX Bruker XFlash 4010 detector.

X-ray diffraction (XRD) analysis was performed with a PANalytical X’Pert PRO diffractometer (Almelo, The Netherlands), using Cu-Kα radiation (0.154187 nm). The diffractometer was operated at 45 kV and 40 mA using a step size of 0.02 and a 500 s exposure time. Phase identification was accomplished by use of search-match software utilizing international center for diffraction data ICDD database (2002, Pennsylvania, USA).

The bioactivity evaluation was performed by soaking the cement disks in 13 mL simulated body fluid (SBF) [23] during 4, 24 and 72 h at 36.5 °C and 60 r.p.m. shaking. Previously to the bioactivity assay, the samples were sterilized under UV light for 10 min period on each side. SBF solution was filtered using 0.2 mm bacteriostatic filter (Biofil).

## 3. Results

### 3.1. Un-Hydrated MTA Repair HP Characterization

**Figure 1:**
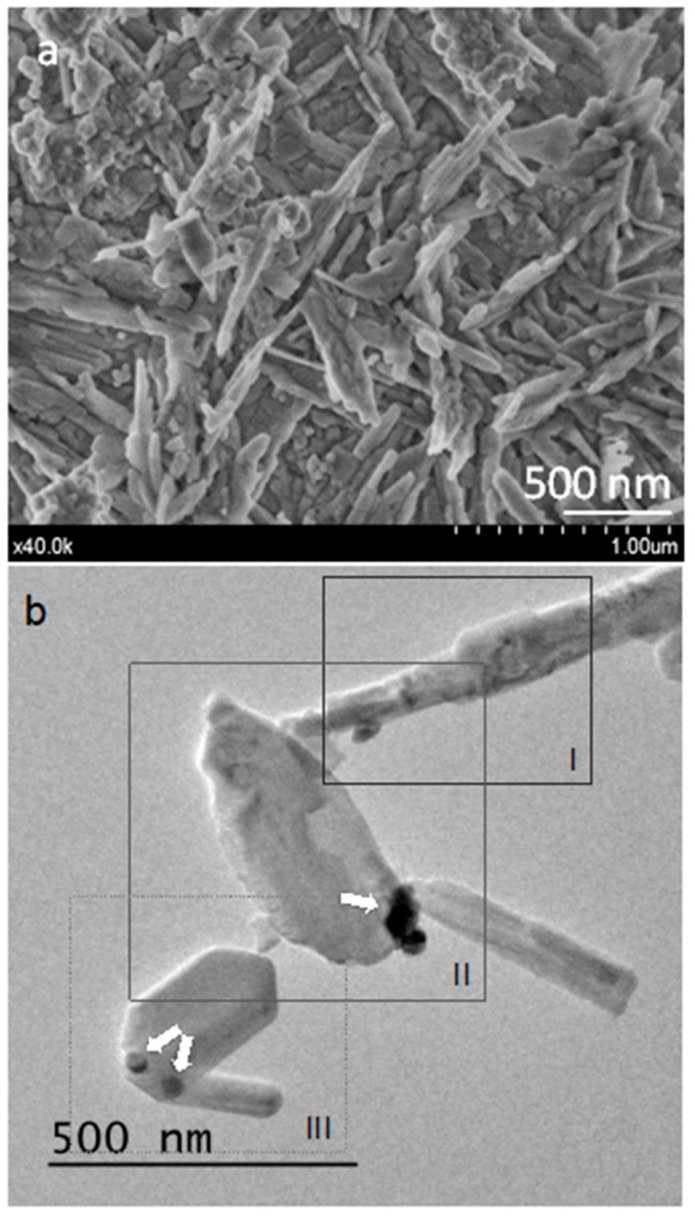
Electron microscopy images of un-hydrated MTA Repair HP material: (**a**) field emission gun scanning electron microscopy (FEG-SEM) secondary electron micrograph; (**b**) TEM bright field image.

FEG-SEM microstructure of un-hydrated powder aggregates showing submicron particles is presented in Figure 1a. Homogeneous needle-like morphologies of 50–100 nm size thicknesses were observed. Powder material dispersion allowed TEM analysis for extending nanoparticle morphology and chemical analysis using EDX. Figure 1b displays a representative bright field image of material nanoparticles. The darker ball shaped features correspond to the highest density tungsten element of the calcium tungstate radiopacifier component. Delineated prismatic nanoparticles (Figure 1b(III)) coexist with others of less-regular shape (Figure 1b(I,II)).

**Figure 2:**
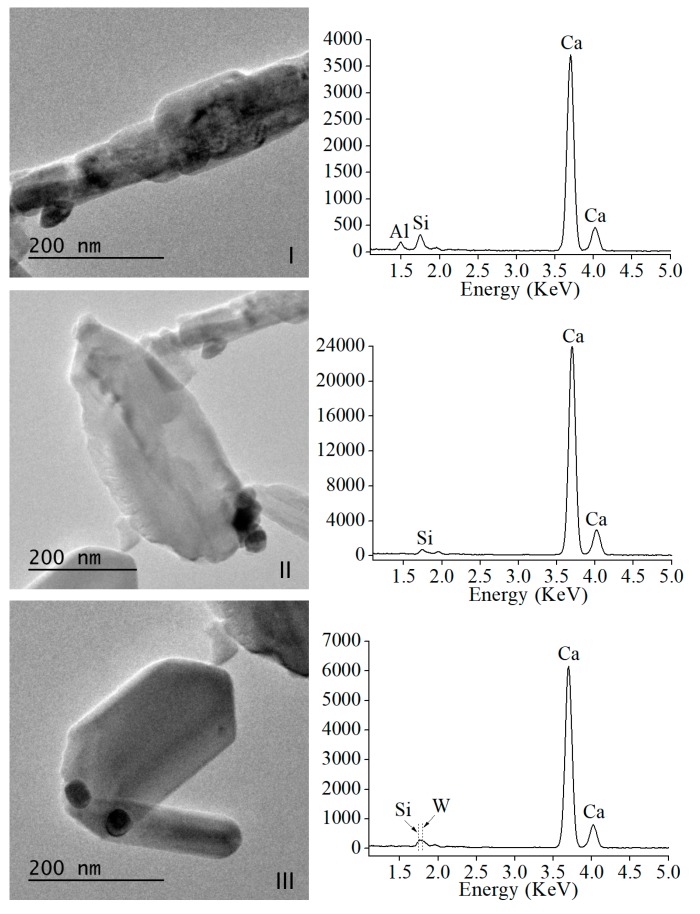
Individual TEM micrographs of un-hydrated MTA Repair HP material taken from (I), (II), and (III) sub-areas of Figure 1b, and corresponding energy dispersive X-ray (EDX) analysis (left column). The y-axis values of EDX plots correspond to acquired energy counts.

The sample area displayed in Figure 1b is further fragmented in three sub-areas, I, II, and III, to preform separate EDX analysis as presented in Figure 2. Ca and Si energies were detected in the three analyzed sub-areas, whilst W in sub-area III only, displaying dark high contrast features. Al energy was only detected in sub-area I, corresponding to the nanoparticle with very irregular morphology in Figure 1b. Moreover, more geometrically regular nanoparticles appear Al free.

**Figure 3:**
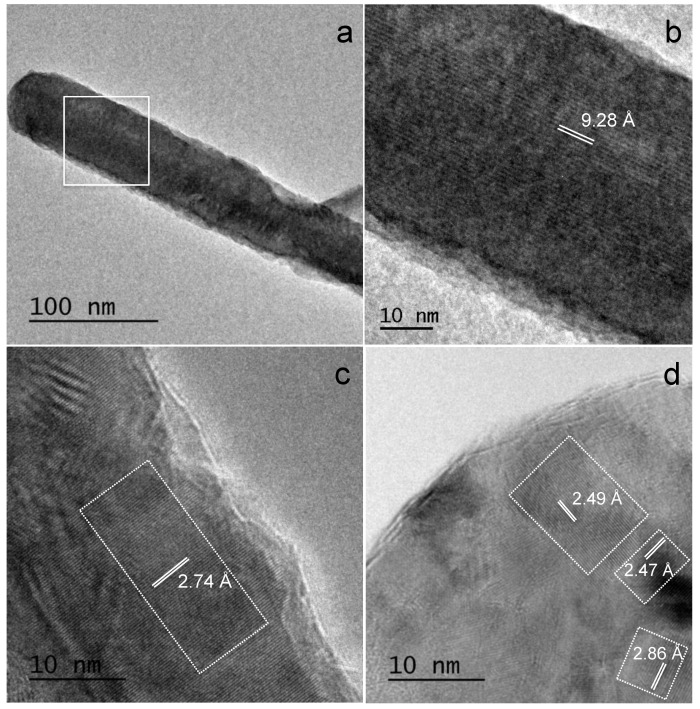
HRTEM images of un-hydrated MTA Repair HP material and d_hkl_ measurements: (**a**) characteristic elongated nanoparticle, and (**b**) magnification of the (**a**) square inset; (**c**) elongated nanoparticle edge and measured nanodomain; (**d**) irregular polycrystalline particle showing different nanodomains.

Representative high-resolution (HR), TEM images, and analyses of the precursor material are displayed in Figure 3. Lattice fringe separation measurements of regularly shaped and elongated MTA Repair HP characteristic nanoparticles displayed in sub-area III, of 9.28 Å (Figure 3a,b), matched only for (002) reflexions of the monoclinic tricalcium silicate phase (powder diffraction file (PDF), 04-019-5754). Other measurements preformed on these elongated nanoparticles of 2.74 Å (Figure 3c) are compatible with both (224) reflexions of tricalcium silicate (PDF 04-019-5754) and (200) reflexions of dicalcium silicate (PDF 01-077-0409). The crystallite lattice fringe separations measured from polycrystalline images, as shown in sub-area I (Figure 3d) of 2.49 Å, matched with dicalcium silicate (PDF 00-009-0351) and with Ca_3_Al_2_O_6_ (PDF 00-032-0148); and 2.47 and 2.86 Å are compatible with tricalcium silicate (PDF 04-019-5754).

### 3.2. Hydrated MTA Repair HP Study

**Figure 4:**
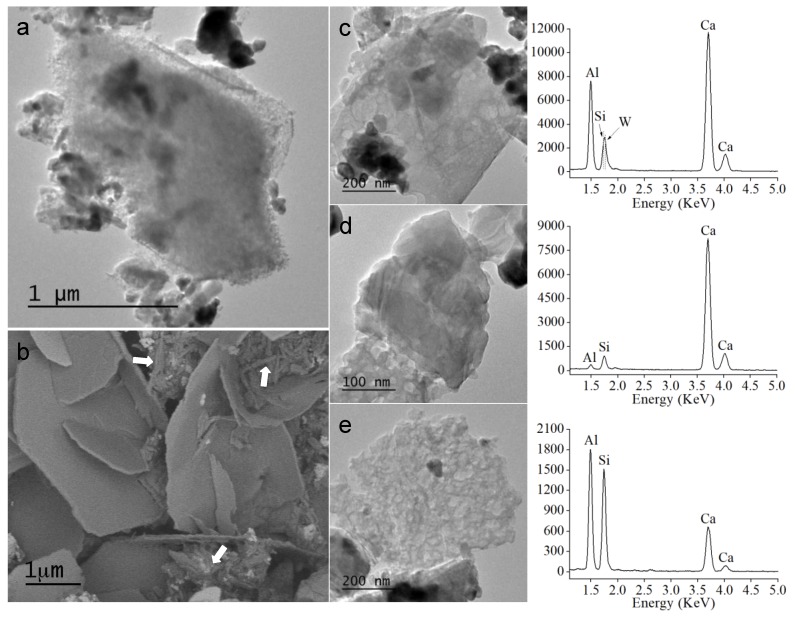
Electron microscopy study of hydrated MTA Repair HP material after 24 h setting: (**a**) TEM and, (**b**) FEG-SEM general view micrographs. White arrows indicate sub-micron needle-like particles; (**c**–**e**) high magnification images showing sample characteristic areas and corresponding EDX analysis (column at the right). The y-axis values of EDX plots correspond to acquired energy counts.

Electron microscopy analysis of 24 h set material is compiled in Figure 4. FEG-SEM micrograph (Figure 4b) shows a representative image of the typical microstructure consisting of plate-like structures with hexagonal shape over 1 µm in size, grown in random directions. Sub-micron needle-like particles distributed along the micron size plates are also observed. TEM micrographs of the material are also presented in Figure 4 and, EDX analyses (right column) of high magnification images selecting sample representative areas (Figure 4c–e). The darker aggregates corresponding to the high intensity contrast of the calcium tungstate phase (Figure 4c) do not present morphological changes with respect to un-hydrated material observations. EDX analyses indicate that dense plate-shaped areas (Figure 4d) have less aluminum content in comparison with other highly porous zones (Figure 4e).

**Figure 5:**
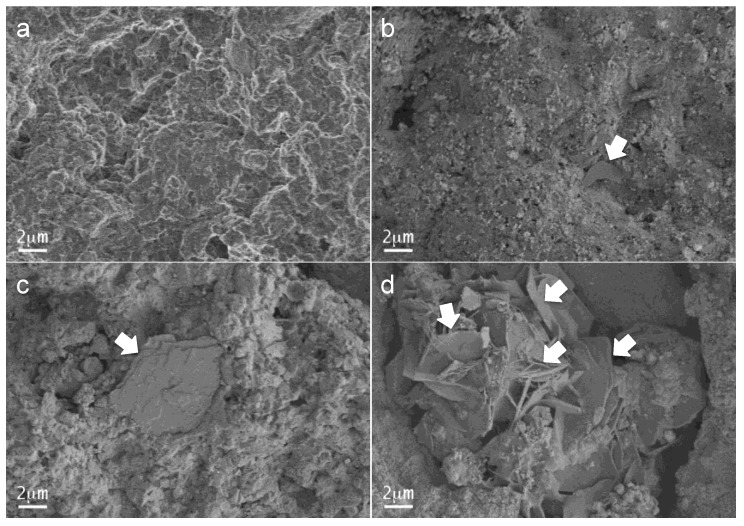
FEG-SEM secondary electron micrographs of hydrated MTA Repair HP material after different setting time period: (**a**) 12 min; (**b**) 4 h; (**c**) 24 h; (**d**) 72 h. White arrows indicate plate-like features observations.

The FEG-SEM observations of MTA Repair HP sample disks after 12 min, 4 h, 24 h, and 72 h setting time intervals are shown in Figure 5. A rough surface of round-shaped forms, observed after the 12 min setting, transformed progressively with time showing increased growth of plate-like grains that spread along the material were highly visible after 72 h.

**Figure 6:**
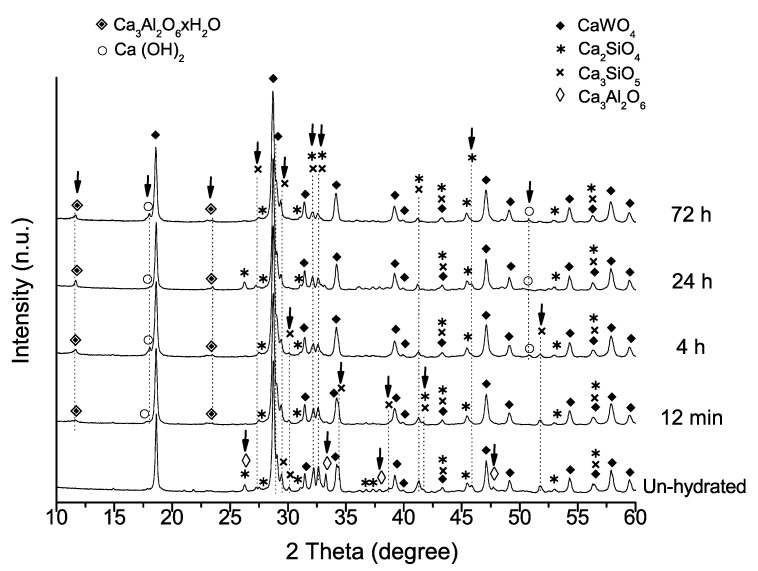
XRD patters of the un-hydrated MTA Repair HP and set material after 12 min, 4 h, 24 h, and 72 h. Black arrows indicate significant variations of detected phases with increasing time of the setting process.

To analyze more of the setting process, XRD patterns of the material after each setting time, are plotted in comparison with the pattern of the un-hydrated sample in Figure 6. Initial un-hydrated powder is composed of tricalcium silicate, Ca_3_SiO_5_ (PDF 01-086-0402), dicalcium silicate, Ca_2_SiO_4_ (PDF 01-077-0409), tricalcium aluminate Ca_3_Al_2_O_6_ (PDF 00-032-0148), and radiopacifier calcium tungstate, CaWO_4_ (PDF 01-077-2233). Intensity peaks assigned to Ca_3_SiO_5_, Ca_2_SiO_4_, and Ca_3_Al_2_O_6_ phases decrease gradually with setting time, while peaks matched with crystalline calcium aluminum oxide hydrate, Ca_3_Al_2_O_6_xH_2_O (PDF 00-002-0083) and Ca(OH)_2_ phases increase.

**Figure 7:**
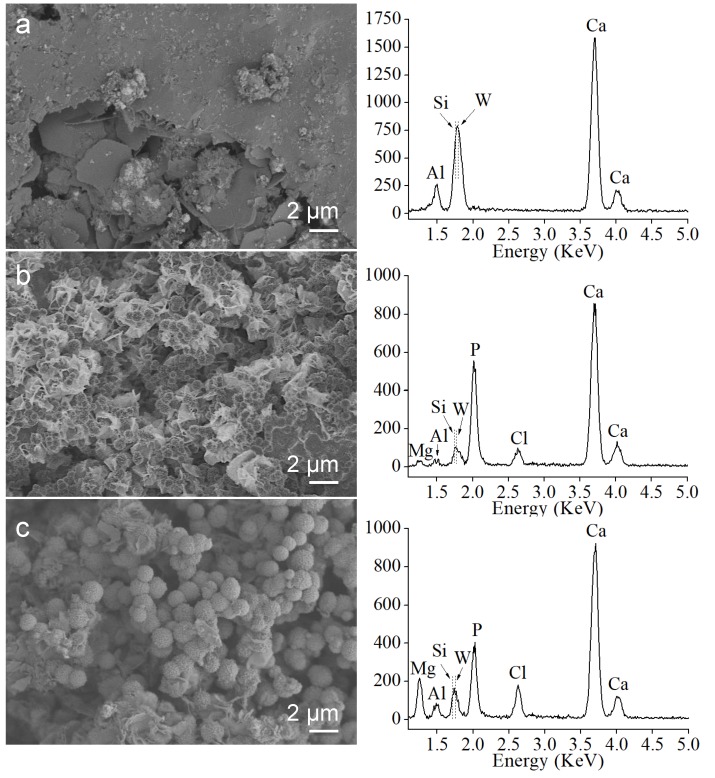
FEG-SEM observations (left) and EDX analysis (right) of the MTA Repair HP set material after simulated body fluid (SBF) bioactivity assay: (**a**) un-treated; (**b**) after 24 h soaking; (**c**) after 72 h soaking. The y-axis values of EDX plots correspond to acquired energy counts.

SBF bioactivity assays using the set material after 24 and 72 h treatment, as well as the un-treated control sample are displayed in Figure 7. After 24 h soaking time, FEG-SEM images indicate a homogeneous surface coating formation by the growth of spherical aggregates covering the surface. Observations after 72 h indicate not only a clear increase in size of individual sphere formations but also the resolution of prismatic features characteristic of calcium apatite-like structure with hexagonal symmetry. Confirmation of surface calcium phosphate deposition is done by EDX analysis (right column in the Figure 7). The magnesium signal detected after 72 h treatment indicates calcium phosphate phase coating incorporation of this element, which is available in the SBF solution. Interestingly, Al, Si, and W relative (to Ca and P) intensity signals from the starting surface components, do not decrease with soaking time within the 24–72 h interval.

**Figure 8:**
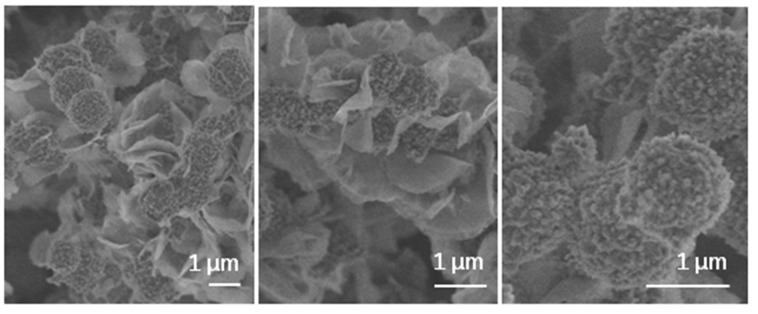
High magnification FEG-SEM secondary electron micrographs of the MTA Repair HP set material after 72 h SBF soaking.

FEG-SEM high magnification images of the typical calcium phosphate formations, growing over characteristic foil-like structures formed on hydrated MTA Repair HP material surface, after 72 h SBF soaking treatment, are presented in Figure 8.

## 4. Discussion

MTA Repair HP dental material is endodontic cement, which shows both a rapid setting rate of 12 min (initial setting time) [21], and a quick and effective bioactive response in vitro in terms of surface coating of calcium phosphate [19]. In this work, a detailed look at the material microstructure highlights particular characteristics to understand these interesting properties.

Electron microscopy analysis of the precursor powder consisting of Ca_3_SiO_5_, Ca_2_SiO_4_, Ca_3_Al_2_O_6_, and radiopacifier component, CaWO_4_, indicates singular sub-micron particle size showing predominantly elongated shape. In relation to this particle size, the relatively low intensity XRD peaks detected for the calcium silicate species of the material in comparison with other calcium silicate cements [21], correlates well with the sub-micron particle sizes observed by FEG-SEM and TEM. The bright field observations using TEM in combination with EDX analysis determined that characteristic MTA Repair HP nanoparticles exhibiting, low contrast imaging, as well as faceted singularity and elongated morphology, do not contain aluminum, indicating that these geometrically regular nanoparticles correspond to a calcium silicate phase. Interestingly, HRTEM analysis of elongated nanoparticles indicates that specific d-spacing measured at 9.28 Å match the (002) reflexions for Ca_3_SiO_5_, whilst no other material component species were compatible with this value. To support the assignation of elongated and faceted nanoparticles to tricalcium silicate, previous work has also described particles with sharp angles typically to Ca_3_SiO_5_ while round-shaped morphologies to Ca_2_SiO_4_ [24].

Tricalcium silicate (Ca_3_SiO_5_) component has been found to be mainly responsible for strength development of hydraulic cements [14]. Ca_3_SiO_5_ reacts with water to form calcium silicate hydrate (CSH), and polymerization of the CSH network contributes to the self-setting properties and increased mechanical strength after aging [25]. Similarly, the dicalcium silicate phase (Ca_2_SiO_4_) reacts with water to form CSH but at a slower rate than Ca_3_SiO_5_ [26]. Both calcium silicates hydration induce the formation of needle-like amorphous CSH gels and columnar-shaped calcium hydroxide crystals [25,27], which intermingle with each other to finally consolidate. Our results will point out that high aspect-ratio Ca_3_SiO_5_ nanometric particles, providing with high surface area of reactivity, could well be an important factor to promote fast silicate hydration reaction [13].

Hydration of calcium silicate cements is affected by the presence of other formulation components, such as calcium aluminate, Ca_3_Al_2_O_6_. The reaction of tricalcium aluminate with water has been reported instantaneous, producing first an amorphous phase that transforms further into hexagonal-plate-like tricalcium aluminate hexahydrate crystals, which harden MTA [27]. MTA Repair HP tricalcium aluminate component fast reaction is corroborated in this XRD study, which shows Ca_3_Al_2_O_6_ peaks disappearing completely after 12 min setting process. The FEG-SEM observations indicate the growth of new micron-size plate-like crystals after 4 h setting, in correlation to the appearance of new XRD peaks at 2θ = 11.6 and 23.6, characteristics of crystalline calcium aluminum oxide hydrate. 

Typically, no peaks corresponding to calcium silicate hydrated products (CSH) were observed by XRD analysis due to poorly crystalline nanoparticles generation, usually described as needle-like, foil or globule formations [25,28]. CSH phase has been mainly based on tobermorite-like structure [28,29], and the proposed nanostructure as a gelled colloid, which enclosed water-filled of internal and interlayer spaces [30]. TEM images displayed in Figure 4 are very illustrative of the complex nano-porosity exhibited by the MTA Repair HP hydrated material. Notably, EDX analysis carried out in especially porous areas indicated a high Al content in relation to Si and Ca elements. Hence, the presence of aluminum in the starting cementitious materials can lead to the precipitation of the aluminum in the CSH phases [29], which could be nourishing for the tricalcium aluminate component in close contact with the high aspect ratio Ca_3_SiO_5_ nanoparticles of the precursor material.

In fact, this work HRTEM analysis reveals that MTA Repair HP distinctive elongated nanoparticles correspond to Ca_3_SiO_5_, which is the main reactive silicate compound of the hydration reaction. DRX analysis carried out at different setting times correlates Ca_3_Al_2_O_6_, major hydration reaction complexion to the actual 12 min initial setting time. We argue, that nanometric size and high aspect ratio of the reactive Ca_3_SiO_5_ component are optimal parameters to promote hydration reaction, not only because of the material high surface area exposed but also for the beneficial close contact to the very reactive Ca_3_Al_2_O_6_. In this respect, presented TEM-EDX analysis of the set material indicates that aluminum ions likely incorporate to the calcium silicate hydrated CSH product.

On the other side, bioactivity, as the capacity of a biomaterial to promote the surface growing of a calcium phosphate layer, which stimulates the biomaterial-tissue interfacial bonding, and tissue regeneration, is determined for the material surface parameters. Both a negative surface charge and a porous substrate have been reported to be required for calcium phosphate formation [31], and enhanced coating formation by the presence of pores between 2 and 50 nm diameter [31,32,33].

SBF experiments carried out of hydrated MTA Repair HP have demonstrated a rapid bioactive response. The hydroxyl group’s rich surface, and the highly nano-porous texture of the foil-like CSH of hydrated MTA Repair HP assessed by TEM are proposed as optimum parameters to stimulate the nucleation and growth of biomimetic calcium phosphate structures. FEG-SEM observations performed after 24 and 72 h SBF soaking show growth of CaP phases upon foil-like hydration morphologies. EDX analysis after SBF treatment indicates that Si energy peak intensity does not decrease in intensity with CaP coating progressing from 24 to 72 h time treatment (Figure 7). These results, in addition to the previous analysis in similar conditions of Si and Ca ionic products release [21], are consistent with further silica-rich layer repolymerization through silanol group’s condensation from the soluble silica.

To summarize, presented results point out that MTA Repair HP precursor material characteristics of tricalcium silicate particle size and high aspect ratio, which provide an elevated surface area and maximized formulation components dispersion of calcium silicate and very reactive calcium aluminate, are key parameters to produce a very effective material in terms of rapid setting capacity and bioactive response.

## 5. Conclusions

HRTEM analysis performed of un-hydrated MTA Repair HP reveals that distinctive high aspect ratio nanoparticles exhibiting both low contrast imaging and geometrically regular morphology correspond to the Ca_3_SiO_5_ phase. DRX analysis carried out of samples using different hydration process times, correlates major Ca_3_Al_2_O_6_ hydration reaction to the actual 12 min initial setting time. Results indicate that the nanometric size and high aspect ratio of the tricalcium silicate particles of MTA HP Repair provide not only an elevated surface area to favor hydration reaction, but also a maximized calcium silicate close contact with the very reactive calcium aluminate component. This combination, of precursor powder nanostructure and formulation, allows a quick hydration process forming a nanoporous calcium aluminate silicate hydrate microstructure, which was effective to achieve both fast setting and efficient bioactive response.
